# Commentary: The Potential Role of the Dipeptidyl Peptidase-4-Like Activity From the Gut Microbiota on the Host Health

**DOI:** 10.3389/fmicb.2018.03313

**Published:** 2019-01-10

**Authors:** Ronivaldo Rodrigues Da Silva

**Affiliations:** Instituto de Biociências, Letras e Ciências Exatas, Universidade Estadual Paulista Júlio de Mesquita Filho (UNESP), São José do Rio Preto, Brazil

**Keywords:** hypothesis, intestinal barrier, microbiota, protease, proteolytic activity

Peptidases or proteases are enzymes essential for various functions of a cell and are present in all living organisms (Ida et al., 2017; Silva, 2018a). They are capable of hydrolyzing the peptide bond (Silva, 2017, 2018b). These can be classified according to (1) the chemical mechanism of catalysis, depending on the nature of their nucleophilic agents; (2) details of the reaction catalyzed, depending on the length of the target substrate and released product, or the location of the peptide bond where the enzyme will act; and (3) molecular structure and homology (Silva, 2017; Merops, 2018^1^).

Dipeptidyl aminopeptidases are enzymes that hydrolyze the penultimate peptide bond at the N-terminus, separating a dipeptide from the remaining substrate (Stressler et al., 2013; Zilleßen et al., 2016). Dipeptidyl peptidase-4 (DPP-4) is an enzyme which exhibits high affinity to proline and alanine in the S_1_ subsite (Merops, 2018). DPP-4 activity can be found in prokaryotes and eukaryotes (Olivares et al., 2018). In humans, these enzymes are present either as a membrane protein or as a soluble enzyme. In some bacteria, it is present as a membrane protein (S9B family). Additionally, a similar type of cytosolic DPP-4 is found in some bacteria, such as *Lactobacillus helveticus* (PepX, S15 family) (Stressler et al., 2013; Merops, 2018; Olivares et al., 2018). DPP-4 activity has also been found in fungi (Olivares et al., 2018).

DPP-4 is present in commensal microorganisms of the human digestive tract anchored to their plasma membranes (Walker et al., 2003). However, very little information currently exists on such microbial enzymes. This subject, which relates to the effect of these enzymes on the host organism, has been addressed in this comment.

This commentary focuses on the work reported by Olivares et al. (2018), in which they have investigated the activity of the DPP-4 present in the gut microbiota of mice, and have hypothesized about its effect on the host organism. The authors base their arguments on the fact that the cecal content of gnotobiotic mice colonized with the gut microbiota of a healthy subject showed increased proteolytic activity of DPP-4 as compared to germ-free mice (GFM). Additionally, cecal tissue mRNA analyzed in both study groups did not demonstrate significant differences in *Dpp-4* gene expression between them. This indicates that significant DPP-4-like activity occurs in the gut microbiota.

Motivated by the results, the authors discuss about the influence of this enzyme on intestinal and hormonal disorders. It is worth emphasizing here that, all arguments being scientifically grounded, what draws attention to the article is the hypothesis of translocation of DPP-4 from the gut microbiota to blood plasma.

The article refers to the work of Marguet et al. (2000), in which *Dpp-4*-knockout (KO) mice exhibited DPP-4 activity in plasma by a hitherto unknown process. Therefore, the authors sought to determine the origin of this enzyme and the mechanism by which it is present in plasma. They hypothesized that DPP-4 is translocated from the gut microbiota through the intestinal wall.

Here, I would like to discuss this hypothesis, in which some points are highlighted: (1) as mentioned by the authors, for the enzyme to be translocated, the microbiota would require to secrete a soluble isoform of the peptidase; (2) the intestinal wall is recognized to be an impermeable barrier to macromolecules, and therefore, increased permeability is associated with intestinal disorder, inflammation, and disease.

As described by the authors, DPP-4 in humans can be found in two isoforms, associated with the plasma membrane or a soluble form in the bloodstream (Matteucci and Giampietro, 2009; Roppongi et al., 2018). In contrast, it has been demonstrated that microbial DPP-4 is either associated with the membrane, or in the case of PepX this enzyme is also present in the intracellular medium. However, it has not yet been verified whether microbes can also secrete DPP-4. Additionally to the active secretion of DPP-4, this enzyme could be also released outside microbial cells by cellular lysis following microbial death in the intestine. This finding would be necessary to support the hypothesis of translocation of the enzyme through the intestinal wall.

Another important aspect to be considered is the permeability of the intestinal wall. The intestinal wall forms a barrier that contributes to absorption of nutrients derived from digested food; however, it does not permit the flow of macromolecules and microorganisms (Fukui, 2016; Thursby and Juge, 2017). A suitable transport membrane system for translocation of these enzymes would be required, without being necessarily accompanied by detrimental effects on the host. Simultaneously, the integrity of the intestinal wall should be maintained. This justifies the need for further research to understand the feasibility of this scientific proposal. These arguments do not, however, disregard the hypothesis raised by Olivares et al. (2018), but only substantiate the need for further investigation.

It is important to note that the discovery of the presence of DPP-4 in the plasma of *Dpp-4* KO mice, and the hypothesis raised by the authors in the attempt to explain this information, is of high interest and would guide scientific discoveries and open a forum for discussion, serving as an avenue for further investigations.

It is well-known that there are differences in primary structure of citosolic PepX (S15 family, E.C. 3.4.14.11) and mammalian DPP-4 (S9B family, E.C. 3.4.14.5) (Olivares et al., 2018). PepX is not found in superior eukaryotic genomes (André et al., 2013). In part, to verify if the dipeptidyl aminopeptidase activity in KO mice for *Dpp-4* gene is a PepX-like, it may possibly be investigated by comparative analysis of the sequence of the enzyme present in the plasma and a gene library of the gut microbiota. Nevertheless, it is quite true that this calls for extensive work! Metagenomics approaches could be useful to validate the presence of this enzyme in the gut microbiota. DNA library construction could be an alternative due to the diversity of the gut microbiota (Gill et al., 2006; Yang et al., 2018). Finding a relationship between the DPP-4 activity present in the plasma of KO mice for *Dpp-4* gene and the enzyme expressed by the gut microbiota is a challenge that may contribute to reinforce the concept of enzyme translocation.

In fact, this work offers support for future investigations. As mentioned by the authors, further research is necessary to validate this hypothesis. The discussion developed by Olivares et al. (2018) is hugely praiseworthy, as in the light of their own results combined with the information currently available in the literature on this subject, it seems possible to associate the gut microbial DPP-4 activity with effects on the host health.

## Author Contributions

The author confirms being the sole contributor of this work and has approved it for publication.

### Conflict of Interest Statement

The author declares that the research was conducted in the absence of any commercial or financial relationships that could be construed as a potential conflict of interest.

## Footnotes

^1^Merops. https://www.ebi.ac.uk/merops/about/classification.shtml#MECHANISM (Accessed December 26, 2018).
